# An Electrochemical Sensor for Diphenylamine Detection Based on Reduced Graphene Oxide/Fe_3_O_4_-Molecularly Imprinted Polymer with 1,4-Butanediyl-3,3′-bis-l-vinylimidazolium Dihexafluorophosphate Ionic Liquid as Cross-Linker

**DOI:** 10.3390/polym10121329

**Published:** 2018-12-01

**Authors:** Lingyu Liu, Xudong Zhu, Yanbo Zeng, Hailong Wang, Yixia Lu, Jian Zhang, Zhengzhi Yin, Zhidong Chen, Yiwen Yang, Lei Li

**Affiliations:** 1 School of Petrochemical Engineering, Changzhou University, Changzhou 213016, China; orsenl@163.com (Lingyu L.); zdchen@cczu.edu.cn (Z.C.); 2 College of Biological, Chemical Sciences and Engineering, Jiaxing University, Jiaxing 314001, China; zhuxudong9zxd@163.com (X.Z.); wanghailong@mail.zjxu.edu.cn (H.W.); luyixia905@163.com (Y.L.); tracychang1983@163.com (J.Z.); yinzhengzhi@mail.zjxu.edu.cn (Z.Y.); yangyiwen@mail.zjxu.edu.cn (Y.Y.)

**Keywords:** reduced graphene oxide, Fe_3_O_4_, molecularly imprinted polymer, ionic liquid cross-linker, electrochemical sensor, diphenylamine

## Abstract

In this paper, we report a new composite of reduced graphene oxide/Fe_3_O_4_-ionic liquid based molecularly imprinted polymer (RGO/Fe_3_O_4_-IL-MIP) fabricated for diphenylamine (DPA) detection. RGO/Fe_3_O_4_-IL-MIP was prepared with RGO/Fe_3_O_4_ as supporter, ionic liquid 1-vinyl-3-butylimidazolium hexafluorophosphate ([VC_4_mim][PF_6_]) as functional monomer, ionic liquid 1,4-butanediyl-3,3’-bis-l-vinylimidazolium dihexafluorophosphate ([V_2_C_4_(mim)_2_][(PF_6_)_2_]) as cross-linker, and diphenylamine (DPA) as template molecule. Fourier transform infrared spectroscopy, thermal gravimetric analysis, scanning electron microscopy, and vibrating sample magnetometer were employed to characterize the RGO/Fe_3_O_4_-IL-MIP composite. RGO/Fe_3_O_4_-IL-MIP was then drop-cast onto a glassy carbon electrode to construct an electrochemical sensor for DPA. The differential pulse voltammetry (DPV) peak current response for 20 μM DPA of RGO/Fe_3_O_4_-IL-MIP modified glassy carbon electrode (GCE) was 3.24 and 1.68 times that of RGO/Fe_3_O_4_-IL-NIP and RGO/Fe_3_O_4_-EGDMA-MIP modified GCEs, respectively, indicating the advantage of RGO/Fe_3_O_4_-IL-MIP based on ionic liquid (IL) as a cross-linker. The RGO/Fe_3_O_4_-IL-MIP sensor demonstrated good recognition for DPA. Under the optimized conditions, the RGO/Fe_3_O_4_-IL-MIP sensor exhibited a DPA detection limit of 0.05 μM (S/N = 3) with a linear range of 0.1–30 μM. Moreover, the new RGO/Fe_3_O_4_-IL-MIP based sensor detected DPA in real samples with satisfactory results.

## 1. Introduction

Diphenylamine (DPA) is used as a pre- or post-harvest scald inhibitor for some fruits, a rubber antioxidant, and a solid fuel rocket propellant [1]. Residues of DPA are found in fruit and environmental water samples [2]. The presence of DPA residues in fruits [3] and the environment pose a hazard to human health since it is classified as a probable human carcinogen. To monitor and control the overuse of this harmful compound, accurate detection of DPA in fruit and environmental water samples is important and desirable. Different analytical methods, such as high-performance liquid chromatography [4], gas chromatography [5], and electrochemical sensor [6] have been explored and reported in literature for the detection of DPA. Among these methods, the electrochemical sensor has gained more interest because of its rapid response, low cost, and high sensitivity.

Molecular imprinting technique is an easy but effective method to develop highly selective, sensitive, and stable molecularly imprinted polymers (MIPs) [7,8,9]. Imprinting mechanism enables molecularly imprinted polymers to selectively recognize target molecules. MIPs offer advantages such as easy synthesis, good chemical and physical stability, cost-effectiveness, and robustness [10]. However, MIPs prepared using the conventional technique are associated with some limitations, such as low binding capacity, high diffusion barrier, and poor selectivity [11]. To overcome the drawbacks of the traditional MIPs, various methods have been developed, including surface imprinting [9], solid-phase synthesis [12], and epitope imprinting [13]. Among these methods, surface imprinting based on creating imprinting cavities onto or near supporting material surfaces has emerged as a promising method, due to the efficient removal of templates, low mass-transfer resistance, and good accessibility to target molecules [9,14]. To achieve high binding capacity, nanosized support materials with a large specific surface area are suitable for preparing surface imprinted polymers [14].

Graphene/Fe_3_O_4_ has gained increasing research interest due to its large surface area, excellent conductivity, and strong magnetic properties [15]. Graphene/Fe_3_O_4_ has been extensively applied in supercapacitor [16], lithium-ion batteries [17], electrochemical detection [18], and drug delivery [19]. In recent years, composites of graphene/Fe_3_O_4_ and molecularly imprinted polymers were prepared using surface imprinting technique to improve selectivity, sensitivity, and shorten binding time for recognizing targets [20]. Wang et al. reported graphene/Fe_3_O_4_ based MIP for the recognition of bovine hemoglobin with acrylamide and *N*,*N*’-methylenebisacrylamide as the functional monomer and cross-linker, respectively [20]. For electrochemical sensing of 17β-estradiol, Zhang et al. prepared graphene/Fe_3_O_4_-MIP using methacrylic acid and divinyl benzene as the functional monomer and cross-linker, respectively [21]. Zhou et al. fabricated a magnetic molecularly imprinted electrochemical sensor of amaranth using graphene/Fe_3_O_4_-MIP membrane using aniline as the electropolymerizable monomer [22]. To further increase electrocatalytic activity and adsorption ability for targets of graphene/Fe_3_O_4_-based MIP, some new functional monomers and cross-linkers can be proposed for incorporation into the MIP framework. Ionic liquids (ILs) are well known for their exceptional physical properties, such as non-volatility, high chemical stability, and superior ionic conductivity [23,24]. ILs as functional monomers for preparing MIPs have been extensively studied [25,26,27,28]. However, few reports of ILs as cross-linkers in molecular imprinting technique are available so far [23,29,30]. To the best of our knowledge, reduced graphene oxide/Fe_3_O_4_-MIP prepared with IL 1,4-butanediyl-3,3’-bis-l-vinylimidazolium dihexafluorophosphate ([V_2_C_4_(mim)_2_][(PF_6_)_2_]) as a cross-linker is yet to be explored. 

Herein we introduce a new composite of RGO/Fe_3_O_4_-IL-MIP prepared using RGO/Fe_3_O_4_ as supporter, IL 1-vinyl-3-butylimidazolium hexafluorophosphate ([VC_4_mim][PF_6_]) as functional monomer, IL [V_2_C_4_(mim)_2_][(PF_6_)_2_] as cross-linker, and DPA as template molecule. The RGO/Fe_3_O_4_-IL-MIP composite was characterized and drop-cast onto a glassy carbon electrode (GCE) to construct an electrochemical sensor for DPA detection. Differential pulse voltammetry (DPV) was employed to investigate the electrochemical behavior of the DPA sensor. The prepared RGO/Fe_3_O_4_-IL-MIP sensor exhibited excellent catalytic activity, high sensitivity, and selectivity for DPA. In addition, the RGO/Fe_3_O_4_-IL-MIP sensor was applied to detect DPA in real samples.

## 2. Materials and Methods

### 2.1. Materials

Ethylene glycol dimethacrylate (EGDMA), 1-vinylimidazole, 4-dibromobutane, nitroaniline, 3-nitroaniline, and 4-nitroaniline were purchased from Sigma-Aldrich (St. Louis, MO, USA). Hydroquinone and catechol were obtained from TCI Co., Ltd. (Tokyo, Japan). Diphenylamine (DPA), 1-naphthylamine, 1,4-phenylenediamine, phenol, and 2,2’-azobisisobutyronitrile (AIBN) were purchased from Aladdin Industrial Corporation (Shanghai, China). Chitosan was obtained from Sinopharm Group Chemical Regent Co., Ltd. (Shanghai, China). RGO/Fe_3_O_4_ (45:55, *w/w*) was purchased from Xianfeng Nanotechnology Co., Ltd. (Nanjing, China). [VC_4_mim][PF_6_] was provided by Lanzhou Institute of Chemical Physics, Chinese Academy of Sciences (Lanzhou, China). [V_2_C_4_(mim)_2_][(PF_6_)_2_] was prepared in our laboratory and characterized by ^1^H NMR and ^13^C NMR. Phosphate buffer was prepared using NaH_2_PO_4_ and Na_2_HPO_4_. Deionized water of 18 MΩ cm was used throughout the experiments. 

### 2.2. Instrumentation

Surface morphology measurements were carried out using a JSM-7500F scanning electron microscope (SEM) (JEOL, Tokyo, Japan). Fourier transform infrared (FT-IR) spectra were recorded on Nicolet Nexus-470 FT-IR spectrometer (Waltham, MA, USA). Thermogravimetric analysis (TGA) was conducted using an STA-449F3 instrument (Netzsch, Selb, Germany). A vibrating sample magnetometer (MPMS3) was used to investigate the magnetic properties of samples. The ^1^HNMR and ^13^CNMR spectra were recorded with a Varian 400-MR spectrometer (Palo Alto, CA, USA). All electrochemical experiments were implemented on a CHI660D electrochemical workstation (CHI Instruments Co., Shanghai, China) with a conventional three electrode system comprising of a platinum wire as the auxiliary electrode, a saturated calomel electrode (SCE) as the reference electrode, and a modified GCE (3 mm diameter) as the working electrode.

### 2.3. Preparation of [V_2_C_4_(mim)_2_][(PF_6_)_2_]

**Scheme 1:**
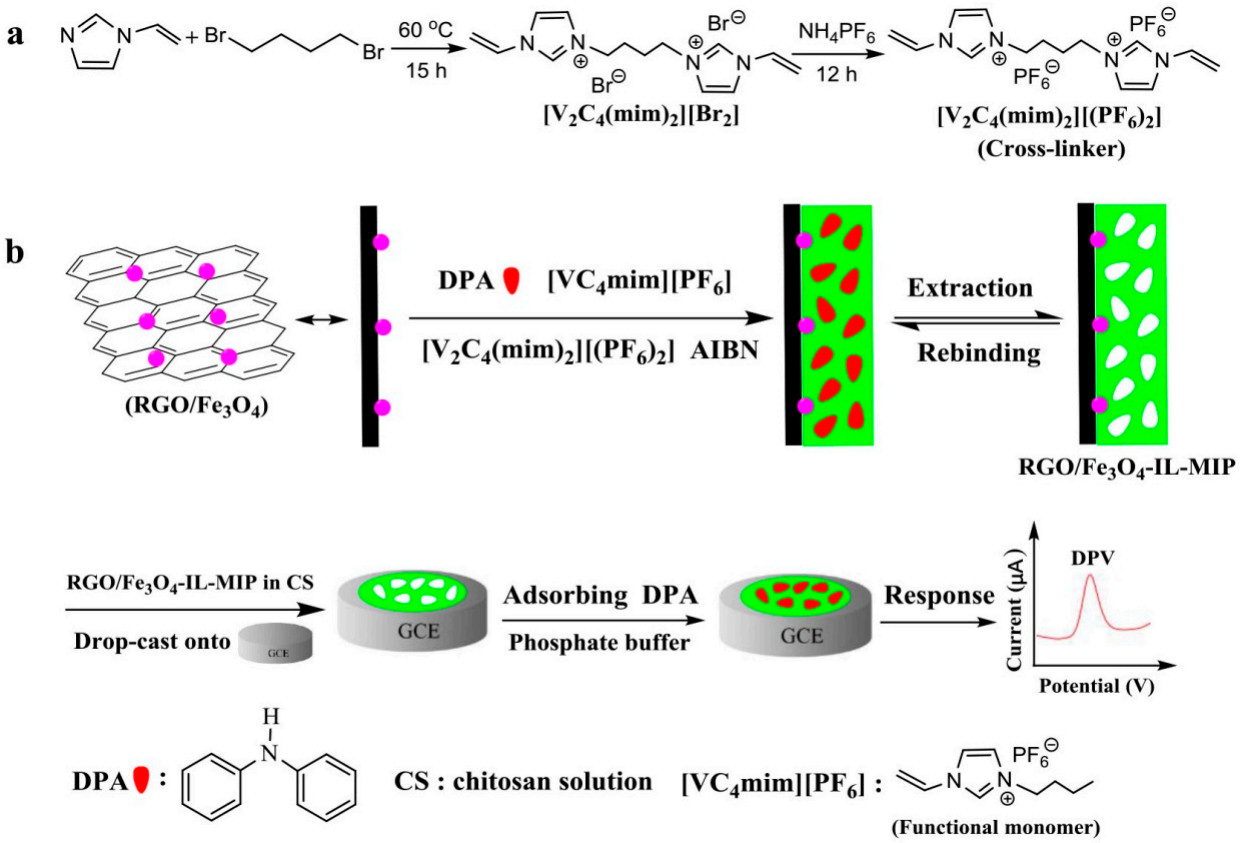
The synthesis route of IL [V_2_C_4_(mim)_2_][(PF_6_)_2_] (**a**). The preparation of RGO/Fe_3_O_4_-IL-MIP and detection process for diphenylamine (DPA) electrochemical sensor (**b**).

Scheme 1a illustrates the synthesis route of the cross-linker [V_2_C_4_(mim)_2_][(PF_6_)_2_]. The synthesis involves two steps. Bromide-based IL [V_2_C_4_(mim)_2_][Br_2_] was first synthesized from 1-vinylimidazole and 1,4-dibromo butane, following the literature methods [23,31]. [V_2_C_4_(mim)_2_][(PF_6_)_2_] was then prepared via an ion-exchange reaction between PF_6_^−^ in NH_4_PF_6_ and Br^−^ in [V_2_C_4_(mim)_2_][Br_2_] [30]. [V_2_C_4_(mim)_2_][Br_2_] (0.05 mol) and NH_4_PF_6_ (0.11 mol) were mixed in water (50 mL) with continuous stirring for 12 h. The product was washed several times thoroughly with water until the product was free of Br^−^, which was detected using AgNO_3_ solution. Finally, [V_2_C_4_(mim)_2_][(PF_6_)_2_] was obtained after vacuum drying overnight at 60 °C. ^1^H NMR (400 MHz, DMSO-d_6_,): δ = 1.82 (s, 4H), 4.21 (s, 4H), 5.41 (d, *J* = 8.8 Hz, 2H), 5.91 (d, *J* = 17.6 Hz, 2H), 7.25 (q, *J* = 8.0 Hz, 2H), 7.86 (s, 2H),8.17 (s, 2H),9.40 (s, 2H); ^13^C NMR (100 MHz, DMSO-d_6_): δ = 26.2 (2C), 48.9 (2C), 109.1 (2C), 119.5 (2C),123.6 (2C), 129.3 (2C), 135.9(2C).

### 2.4. Synthesis of RGO/Fe_3_O_4_-IL-MIP

RGO/Fe_3_O_4_ (0.06 g), DPA (0.05 mmol), and [VC_4_mim][PF_6_] (0.2 mmol) were dispersed in mixed solvent containing acetonitrile and toluene (30 mL, *V*_acetonitrile_:*V*_toluene_ = 1:1). [V_2_C_4_(mim)_2_][(PF_6_)_2_] (1.0 mmol) and AIBN (50 mg) were added into the mixed solution. This solution was purged with nitrogen for 30 min. The temperature was increased to 60 °C, and the reaction solution was allowed to polymerize for 24 h. The polymerized product of RGO/Fe_3_O_4_-IL-MIP was washed thoroughly with methanol-acetic acid solution (*V*_methanol_/*V*_acetic acid_ = 9:1) to extract the template molecule. The non-imprinted polymer (RGO/Fe_3_O_4_-IL-NIP) was synthesized following the same procedure in absence of the template molecule in the polymerization process. To compare the performance of RGO/Fe_3_O_4_-IL-MIP with a traditional cross-linker EGDMA based polymer, RGO/Fe_3_O_4_-EGDMA-MIP was prepared following the same procedure of RGO/Fe_3_O_4_-IL-MIP, replacing [V_2_C_4_(mim)_2_][(PF_6_)_2_] by EGDMA (The amount of EGDMA was 1.0 mmol). 

### 2.5. Electrochemical Measurements

Scheme 1b displays the DPA detection process using the new RGO/Fe_3_O_4_-IL-MIP electrochemical sensor. RGO/Fe_3_O_4_-IL-MIP or RGO/Fe_3_O_4_-IL-NIP (5.0 mg) was dispersed in HAc (1 mL, 1 M) containing chitosan (0.5 wt %) by ultrasonication for 30 min. Each suspension (5.0 μL) was drop-cast on the surface of the cleaned GCE and dried under an infrared lamp. The modified electrodes were incubated in solutions (10 mL) containing different concentrations of DPA in phosphate buffer (0.1 M, pH 5.0) for 4 min and measured by DPV from 0.2 to 1.0 V. The pulse amplitude, pulse period, and pulse width of DPV were 50 mV, 0.25 s, and 50 ms, respectively.

## 3. Results

### 3.1. Characterization of RGO/Fe_3_O_4_-IL-MIP

**Figure 1:**
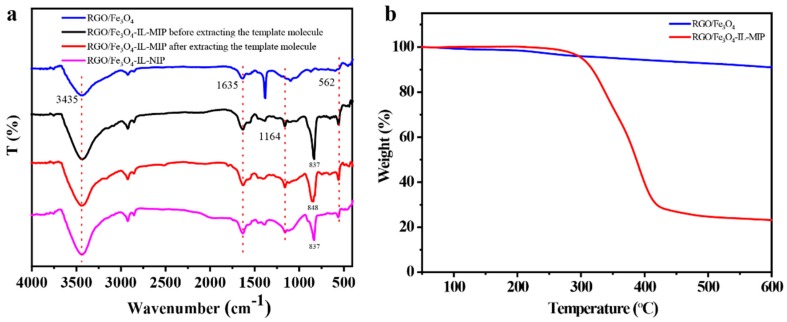
(**a**) FT-IR spectra of RGO/Fe_3_O_4_, RGO/Fe_3_O_4_-IL-MIP before and after extracting the template molecule, and RGO/Fe_3_O_4_-IL-NIP. (**b**) Thermogravimetric analysis (TGA) curves of RGO/Fe_3_O_4_ and RGO/Fe_3_O_4_-IL-MIP.

FT-IR spectra of RGO/Fe_3_O_4_, RGO/Fe_3_O_4_-IL-MIP before and after extracting the template molecule, and RGO/Fe_3_O_4_-IL-NIP are shown in Figure 1a. The peaks at 3435 and 1635 cm^−1^ in the spectrum of RGO/Fe_3_O_4_ are assigned to the stretching vibrations of O–H and C=C [14,32], respectively. The peak at 562 cm^−1^ for RGO/Fe_3_O_4_ corresponds to the stretching vibration of Fe–O of Fe_3_O_4_ [33]. While comparing the spectra of RGO/Fe_3_O_4_-IL-MIP before extracting the template molecule with RGO/Fe_3_O_4_, peaks at 1164 and 837 cm^−1^ were observed, corresponding to the imidazolium cation vibration of the C–N bond and the P–F of PF_6_^−^ stretching vibration [34], respectively. The results suggest the functionalization of PF_6_^−^ based imidazolium ILs onto RGO/Fe_3_O_4_. The spectra of RGO/Fe_3_O_4_-IL-MIP before and after extracting the template molecule and RGO/Fe_3_O_4_-IL-NIP are similar. It is worthy to point out that DPA peaks are not obvious from the spectra of RGO/Fe_3_O_4_-IL-MIP before extracting the template molecule. This phenomenon may be explained by the fact that the DPA peaks were covered by RGO/Fe_3_O_4_-IL-MIP before extracting the template molecule. These results demonstrate the successful synthesis of RGO/Fe_3_O_4_-IL-MIP. Figure 1b displays the TGA curves of RGO/Fe_3_O_4_ and RGO/Fe_3_O_4_-IL-MIP. While a small weight loss of about 8.9% at 600 °C was noted for RGO/Fe_3_O_4_ owing to the presence of oxygen groups on RGO/Fe_3_O_4_, a total weight loss of 76.8% for RGO/Fe_3_O_4_-IL-MIP at the same temperature was recorded, likely due to the thermal instability of MIP. Different thermal stabilities shown by RGO/Fe_3_O_4_ and RGO/Fe_3_O_4_-IL-MIP confirm formation of MIP on the RGO/Fe_3_O_4_ surface.

**Figure 2:**
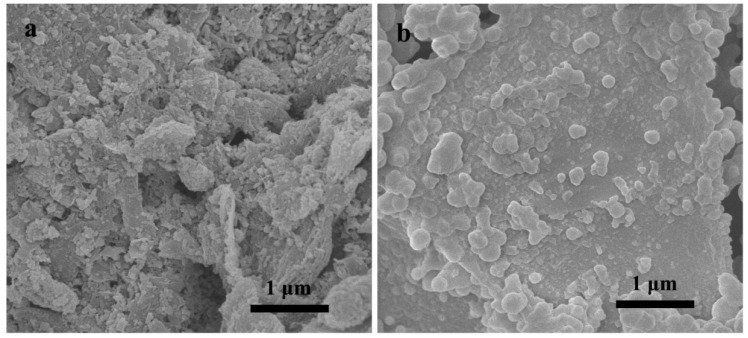
SEM of RGO/Fe_3_O_4_ (**a**) and RGO/Fe_3_O_4_-IL-MIP (**b**).

**Figure 3:**
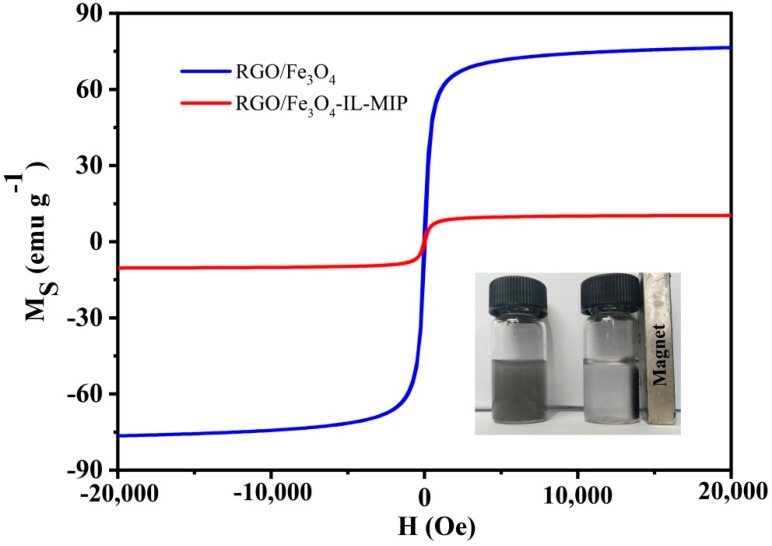
The magnetic hysteresis loop of RGO/Fe_3_O_4_ and RGO/Fe_3_O_4_-IL-MIP. Inset: The magnetic response of RGO/ Fe_3_O_4_-IL-MIP to an external magnetic field.

The surface morphologies of RGO/Fe_3_O_4_ and RGO/Fe_3_O_4_-IL-MIP were characterized by SEM (Figure 2). Apart from the wrinkled layers, distribution of Fe_3_O_4_ nanoparticles on the basal planes of graphene is also evident in the SEM micrograph of RGO/Fe_3_O_4_. In contrast, the surface of RGO/Fe_3_O_4_-IL-MIP is monolithic with a solid base of polymeric material, thus confirming the formation of MIP layer onto the surface of RGO/Fe_3_O_4_. Figure 3 displays the magnetic properties of RGO/Fe_3_O_4_ and RGO/Fe_3_O_4_-IL-MIP along with the dispersion and agglomeration processes of RGO/Fe_3_O_4_-IL-MIP in the inset picture. The saturation magnetizations of RGO/Fe_3_O_4_ and RGO/Fe_3_O_4_-IL-MIP are 76.5 and 10.4 emu·g^−1^, respectively. We attributed this decrease in saturation magnetization for RGO/Fe_3_O_4_-IL-MIP to the presence of MIP layer onto the RGO/Fe_3_O_4_ surface. The saturation magnetization value of RGO/Fe_3_O_4_-IL-MIP was sufficient to ensure easy separation of RGO/Fe_3_O_4_-IL-MIP from a solution by a magnet, as evident in the inset picture of Figure 3. 

### 3.2. Electrochemical Behavior of RGO/Fe_3_O_4_-IL-MIP/GCE

**Figure 4:**
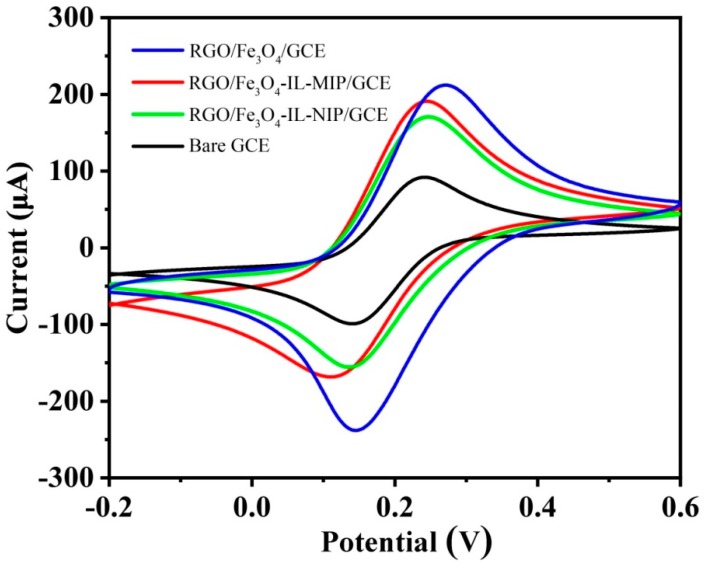
Cyclic voltammograms of RGO/Fe_3_O_4_/GCE, RGO/Fe_3_O_4_-IL-MIP/GCE, RGO/Fe_3_O_4_-IL-NIP/GCE and bare glassy carbon electrode (GCE) in 5.0 mM [Fe(CN)_6_]^3−/4−^ solution containing 0.1 M KCl. Scan rate was 100 mV·s^−1^.

To investigate the electrochemical behavior of RGO/Fe_3_O_4_-IL-MIP, we performed cyclic voltammetry (CV) experiments in 5 mM [Fe(CN)_6_]^3−/4−^ solution containing 0.1 M KCl (Figure 4). Bare GCE exhibited the lowest peak currents, while we observed the highest peak currents for RGO/Fe_3_O_4_/GCE, ascribed to the good electrocatalytic property of RGO/Fe_3_O_4_. The result also indicates the advantage of RGO/Fe_3_O_4_ as supporter. We observed higher peak currents for RGO/Fe_3_O_4_-IL-MIP/GCE compared with that of RGO/Fe_3_O_4_-IL-NIP/GCE. We attribute the higher peak current exhibited by the MIP to the cavities, as [Fe(CN)_6_]^3−/4−^ could pass through these cavities and reach the surface of the electrode more easily.

**Figure 5:**
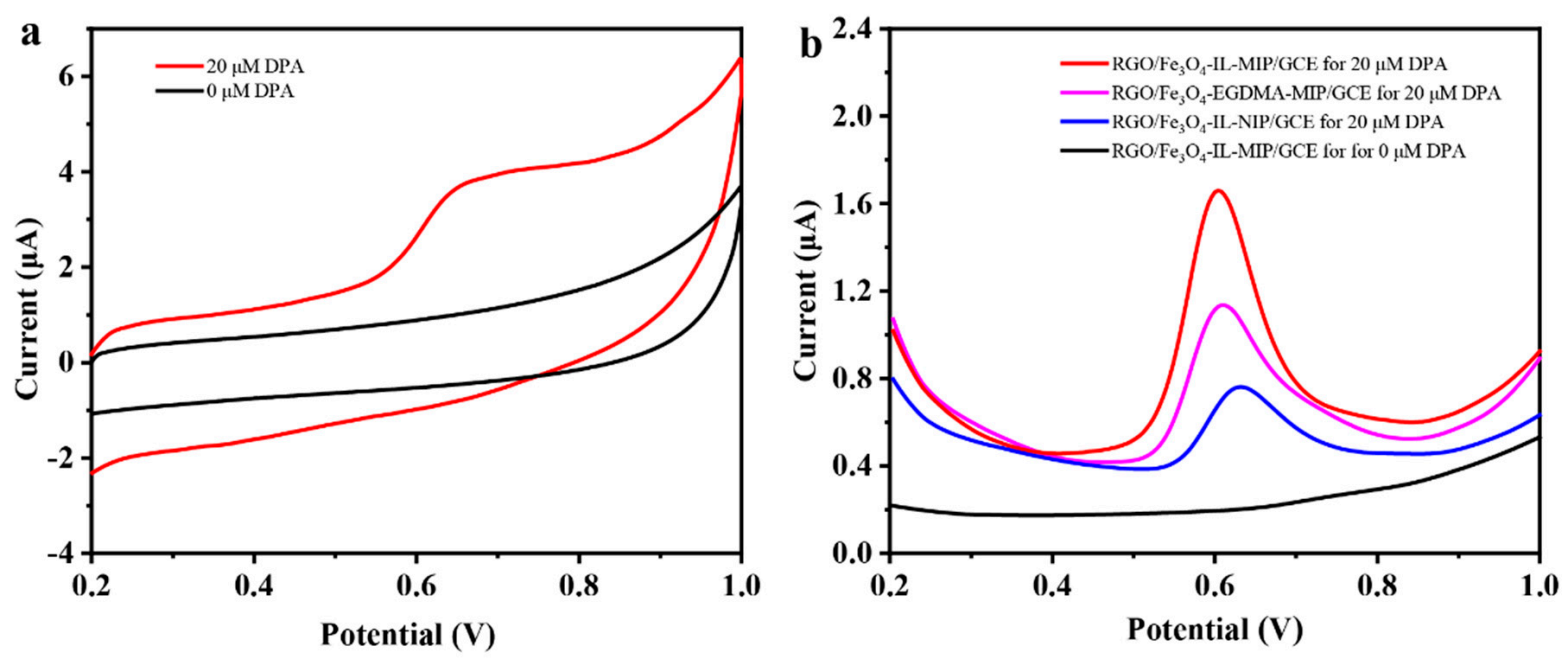
(**a**) Cyclic voltammogram (CV) curve of RGO/Fe_3_O_4_-IL-MIP/GCE after incubating in 0 and 20 μM DPA for 4 min. Scan rate was 100 mV·s^−1^. (**b**) Differential pulse voltammetry (DPV) curves of RGO/Fe_3_O_4_-EGDMA-MIP/GCE and RGO/Fe_3_O_4_-IL-NIP/GCE after incubating in 20 μM DPA solution for 4 min; DPV curves of RGO/Fe_3_O_4_-IL-MIP/GCE after incubating in 0 and 20 μM DPA solution for 4 min.

We studied the electrochemical behavior of 0 μM DPA on RGO/Fe_3_O_4_-IL-MIP/GCE using CV and DPV. There was no peak of CV and DPV observed at the RGO/Fe_3_O_4_-IL-MIP/GCE in 0.1 M phosphate buffer. We investigated the electrochemical behavior of 20 μM DPA on RGO/Fe_3_O_4_-IL-MIP/GCE using CV and observed an oxidation peak at 0.657 V without any reduction peak between 0.2 and 1.0 V, indicating DPA oxidation is an irreversible process [35,36] (Figure 5a). To examine the electrochemical responses for DPA, RGO/Fe_3_O_4_-IL-MIP/GCE and RGO/Fe_3_O_4_-IL-NIP/GCE were incubated in 20 μM DPA solution in phosphate buffer (0.1 M, pH 5.0) for 4 min, and the responses were measured by DPV (Figure 5b). RGO/Fe_3_O_4_-IL-MIP/GCE showed higher DPV peak current response for DPA than RGO/Fe_3_O_4_-IL-NIP/GCE. The DPV peak current response for 20 μM DPA of RGO/Fe_3_O_4_-IL-MIP/GCE was 3.24 times that of RGO/Fe_3_O_4_-IL-NIP/GCE. We again attribute this higher current response of RGO/Fe_3_O_4_-IL-MIP to the imprinted cavities. In addition, the DPV peak current response for 20 μM DPA of RGO/Fe_3_O_4_-IL-MIP/GCE was 1.68 times that of RGO/Fe_3_O_4_-EGDMA-MIP/GCE, indicating the superiority of RGO/Fe_3_O_4_-IL-MIP based on IL as a cross-linker.

### 3.3. Optimization of Experimental Conditions

**Figure 6:**
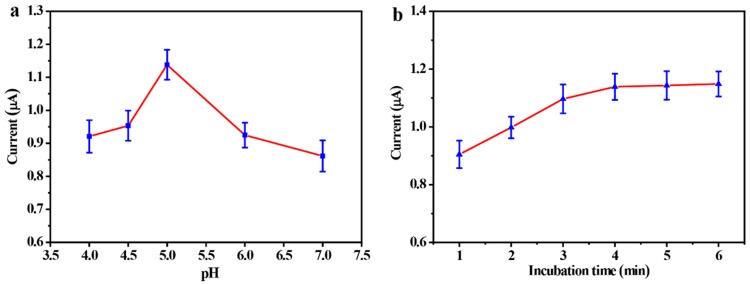
The effect of detection solution pH (**a**) and incubation time (**b**) on DPV peak currents at RGO/Fe_3_O_4_-IL-MIP/GCE, after incubating in 20 μM DPA in phosphate buffer (0.1 M) (*n* = 3).

**Figure 7:**
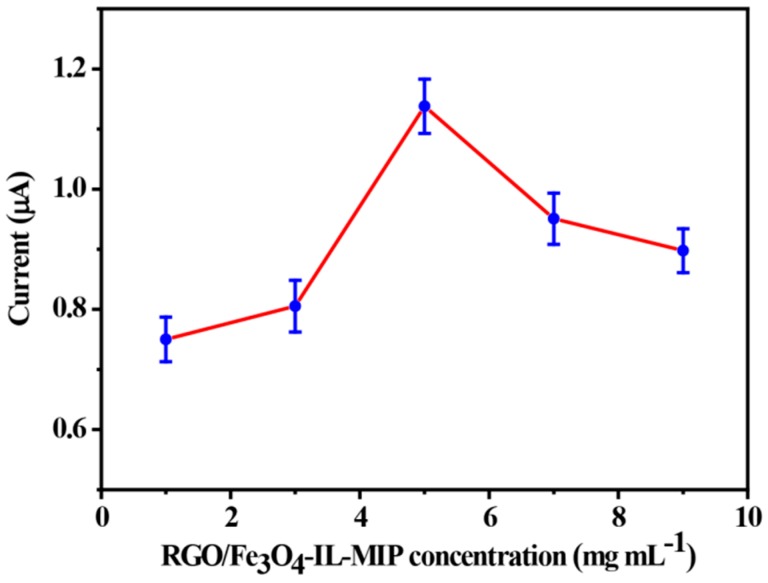
The effect of RGO/Fe_3_O_4_-MIP concentration on DPV peak currents at RGO/Fe_3_O_4_-IL-MIP/GCE, after incubating in 20 μM DPA in phosphate buffer (0.1 M, pH 5.0) (*n* = 3).

Optimization of experimental conditions is important to achieve maximum effect by any system. We optimized the following experimental parameters for DPA detection: pH of the incubation solution, incubation time, and the concentration of RGO/Fe_3_O_4_-IL-MIP. While studying the influence of the incubation solution pH on DPV peak current of DPA, we observed a gradual increase in the oxidation peak current alongside an increase in pH from 4.0 to 5.0, which later decreased with a further increase in pH from 5.0 to 7.0 (Figure 6a). Hence, we selected pH 5.0 as the optimum pH for further experiments. Figure 6b shows a sharp increase in the peak current within the first 4 min before reaching a plateau. Based on this, we selected 4 min as the incubation time for all subsequent experiments. With an increase in the concentration of RGO/Fe_3_O_4_-IL-MIP in HAc (1 M) containing chitosan (0.5 wt %) from 1.0 to 9.0 mg·mL^−1^, the DPV peak current for DPA increased (Figure 7). We attribute this increase in the peak current to the expansion of the conductive electrode area to accumulate more DPA. When RGO/Fe_3_O_4_-IL-MIP concentration reached beyond 5.0 mg·mL^−1^, the peak current decreased. This decrease can be explained in terms of the thickness of RGO/Fe_3_O_4_-IL-MIP restricting the transfer of DPA molecules to the GCE surface. Hence, we chose a concentration of 5.0 mg·mL^−1^ for RGO/Fe_3_O_4_-IL-MIP as the optimized value to construct our DPA sensor.

### 3.4. Analytical Performance and Selectivity of RGO/Fe_3_O_4_-IL-MIP Sensor

**Figure 8:**
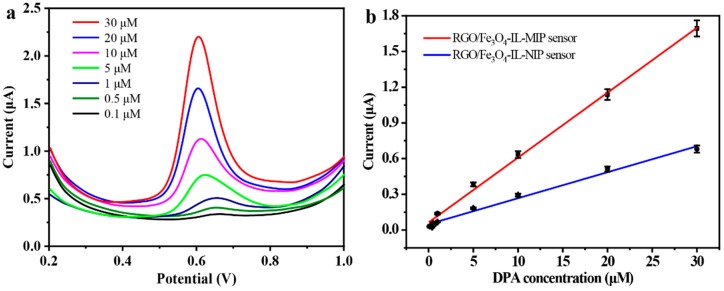
(**a**) DPV curves of RGO/Fe_3_O_4_-IL-MIP sensor for DPA from 0.1 to 30 μM. (**b**) The calibration plots for DPA by RGO/Fe_3_O_4_-IL-MIP and RGO/Fe_3_O_4_-IL-NIP sensors (*n* = 3).

Under the optimized conditions, we examined the detection performance of the RGO/Fe_3_O_4_-IL-MIP/GCE (RGO/Fe_3_O_4_-IL-MIP sensor) towards DPA using DPV. Figure 8a shows a gradual increase in the oxidation peak currents of DPA with increasing DPA concentration, and the oxidation peak currents display a good linear dependence on DPA concentrations in the range of 0.1–30 μM (Figure 8b). The following linear regression equation was used with a correlation coefficient R of 0.9982: I (μA) = 0.0531C (μM) + 0.0687. The sensor achieved a detection limit of 0.05 μM (S/N = 3) for DPA. We also investigated the linearity of RGO/Fe_3_O_4_-IL-NIP sensor for DPA (Figure 8b). The regression equation of RGO/Fe_3_O_4_-IL-NIP sensor for DPA was I (μA) = 0.0218 C (μM) + 0.0486 (R = 0.9950). The RGO/Fe_3_O_4_-IL-NIP sensor had a linear range over DPA concentration from 0.4 to 30 μM. K_1_ and K_2_ are the linear slopes of RGO/Fe_3_O_4_-IL-MIP and RGO/Fe_3_O_4_-IL-NIP sensors, respectively. The K_1_/K_2_ is 2.4, showing that the RGO/Fe_3_O_4_-IL-MIP sensor has a higher peak current than RGO/Fe_3_O_4_-IL-NIP. This result is attributed to the specific cavities of RGO/Fe_3_O_4_-IL-MIP. 

**Figure 9:**
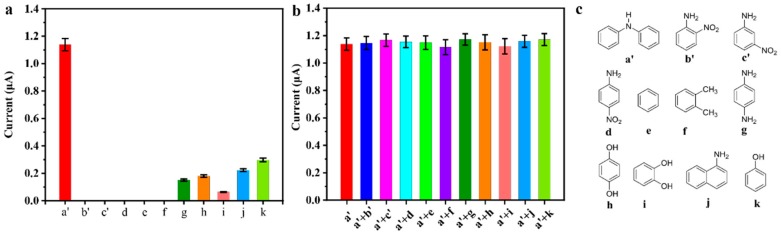
(**a**) DPV peak current response of RGO/Fe_3_O_4_-IL-MIP sensor after incubating in 20 μM DPA and its analogs (*n* = 3). (**b**) DPV peak current response of RGO/Fe_3_O_4_-IL-MIP sensor after incubating in 20 μM DPA and 20 μM DPA in the presence of other analogs (*n* = 3). (**c**) The chemical structures of DPA (a’) and its analogs of 2-nitroaniline (b’), 3-nitroaniline (c’), 4-nitroaniline (d), benzene (e), 1,2-dimethylbenzene (f), 1,4-phenylenediamine (g), hydroquinone (h), catechol (i), 1-naphthylamine (j), and phenol (k).

To evaluate the selectivity of the RGO/Fe_3_O_4_-IL-MIP sensor, we studied the electrochemical responses of 20 μM DPA, 20 μM DPA analogs, and 20 μM DPA in the presence of some analogs. Figure 9a shows that the DPV peak current responses of the RGO/Fe_3_O_4_-IL-MIP sensor for DPA (a’) are higher than other analogs (b’, c’, from d to k, DPV peak current responses were obtained from 0.2 to 1.0 V). In addition, we did not find any interferences for detecting 20 μM DPA (DPV signal change < 5%) in the presence of 10-fold 2-nitroaniline (b’), 3-nitroaniline (c’), 4-nitroaniline (d), benzene (e), 1,2-dimethylbenzene (f), 5-fold 1,4-phenylenediamine (g), hydroquinone (h), 1-fold catechol (i), 1-naphthylamine (j), and phenol (k) (Figure 9b). Figure 9c shows the chemical structures of DPA and its analogs. The results suggest good imprinting efficacy achieved by RGO/Fe_3_O_4_-IL-MIP composites, enabling specific recognition ability towards the template DPA. 

Table 1 summarizes a comparative list of the results of DPA detection by RGO/Fe_3_O_4_-IL-MIP/GCE and other published electrochemical methods [6,37,38,39]. The comparative results indicate that RGO/Fe_3_O_4_-IL-MIP/GCE with MIP material exhibits a low detection limit and high selectivity, which are attributed to the combined effect of good conductivity and imprinting effect. 

### 3.5. Reproducibility and Stability of RGO/Fe_3_O_4_-IL-MIP Sensor

Reproducibility and stability are the two important parameters for applicability of any sensor. To examine the reproducibility of the imprinted sensor, we studied the current responses of DPA solution (20 μM) six times using the same RGO/Fe_3_O_4_-IL-MIP sensor. The current response showed a relative standard deviation (RSD) of 4.1%. Furthermore, after storing for two weeks in a refrigerator, the RGO/Fe_3_O_4_-IL-MIP sensor retained 94.7% of its initial current response for DPA (20 μM). These results certainly indicate the potential applicability of RGO/Fe_3_O_4_-IL-MIP sensor in DPA detection.

### 3.6. Analytical Application

To evaluate the feasibility of the RGO/Fe_3_O_4_-IL-MIP sensor towards the detection of DPA in real samples, we collected lake water samples from Jiaxing University and filtered through 0.45 μm filters. We also prepared food samples including pear and apple peels by grinding them to slurries followed by centrifuging to obtain supernatants. No DPA was detected in those real samples by the RGO/Fe_3_O_4_-IL-MIP sensor. Therefore, we employed the spiking method to demonstrate DPA detection by the RGO/Fe_3_O_4_-IL-MIP sensor. As summarized in Table 2, the recoveries of detection results ranged from 95.6% to 115.2%, demonstrating the reliable detection of DPA in water samples by the RGO/Fe_3_O_4_-IL-MIP sensor.

## 4. Conclusions

A new RGO/Fe_3_O_4_-IL-MIP composite was prepared using RGO/Fe_3_O_4_ as supporter, [VC_4_mim][PF_6_] as functional monomer, [V_2_C_4_(mim)_2_][(PF_6_)_2_] as cross-linker, and DPA as template molecule. The RGO/Fe_3_O_4_-IL-MIP composite was drop-cast onto GCE to construct an electrochemical sensor for DPA detection. The RGO/Fe_3_O_4_-IL-MIP sensor had a higher DPV peak current response than that of RGO/Fe_3_O_4_-EGDMA-MIP, indicating the superiority of RGO/Fe_3_O_4_-IL-MIP based on IL as cross-linker. The detection limit of this sensor reaches 0.05 μM with a wide linear range of 0.1–30 μM. The RGO/Fe_3_O_4_-IL-MIP sensor possesses good selectivity for DPA compared with other analogs, and was used for DPA in real samples in this study with satisfactory results.

## Figures and Tables

**Table 1:** Comparison with other electrochemical methods for the determination of DPA.

Detection Method	Selective Material	Linear Range (μM)	Detection Limit (μM)	Ref.
PEDOT ^a^/MIP/gold electrode	EGDMA ^b^-based MIP	4.95–115	3.9	[6]
EuMoSe_2_/GCE	NR ^c^	0.01–243.17	0.0088	[37]
SrMoO_4_/GCE	NR	0.1–35	0.03	[38]
MIP-based CPE ^d^	TRIM ^e^-based MIP	500–3000	100	[39]
RGO/Fe_3_O_4_-MIP/GCE	IL-based RGO/Fe_3_O_4_-MIP	0.1–30	0.05	This work

^a^ Poly(3,4-ethylenedioxythiophene); ^b^ Ethylene glycol dimethacrylate; ^c^ Not reported; ^d^ Carbon paste electrode; ^e^ Trimethylolpropane trimethacrylate.

**Table 2:** Determination of DPA in samples with the RGO/Fe_3_O_4_-MIP sensor (*n* = 3).

Sample	Added DPA (μM)	Found DPA (μM)	Recovery (%)	RSD (%)
Lake water	1.0	1.039	103.9	3.6
	5.0	4.78	95.6	4.8
Pear peel	1.0	0.993	99.3	4.6
	5.0	5.76	115.2	2.5
Apple peel	1.0	1.15	115.0	4.5
	5.0	5.32	106.4	4.7
